# Widespread Disease in Hedgehogs (*Erinaceus europaeus*) Caused by Toxigenic *Corynebacterium ulcerans*

**DOI:** 10.3201/eid2710.203335

**Published:** 2021-10

**Authors:** An Martel, Filip Boyen, Jörg Rau, Tobias Eisenberg, Andreas Sing, Anja Berger, Koen Chiers, Sarah Van Praet, Serge Verbanck, Muriel Vervaeke, Frank Pasmans

**Affiliations:** Ghent University, Merelbeke, Belgium (A. Martel, F. Boyen, K. Chiers, S. Van Praet, S. Verbanck, F. Pasmans);; Chemical and Veterinary Analysis Agency Stuttgart, Fellbach, Germany (J. Rau);; Hessian State Laboratory, Giessen, Germany (T. Eisenberg);; Bavarian Health and Food Safety Authority, Oberschleißheim, Germany (A. Sing, A. Berger);; Agency for Nature and Forests, Brussels, Belgium (M. Vervaeke)

**Keywords:** Corynebacterium ulcerans, bacteria, diphtheria, hedgehogs, Erinaceus europaeus, disease, toxigenic, antimicrobial resistance, zoonoses, Flanders, Belgium

## Abstract

Toxin-producing *Corynebacterium ulcerans*, a causative agent of diphtheria in humans, was isolated from 53 hedgehogs in Belgium during the spring of 2020. Isolates showed low levels of acquired antimicrobial drug resistance. Strain diversity suggests emergence from an endemic situation. These findings stress the need for raising public awareness and improved wildlife disease surveillance.

Hedgehogs across northern Belgium are currently being affected by an ulcerative skin disease. The purpose of this study was to identify the cause of these skin lesions. 

## The Study 

**Figure 1:**
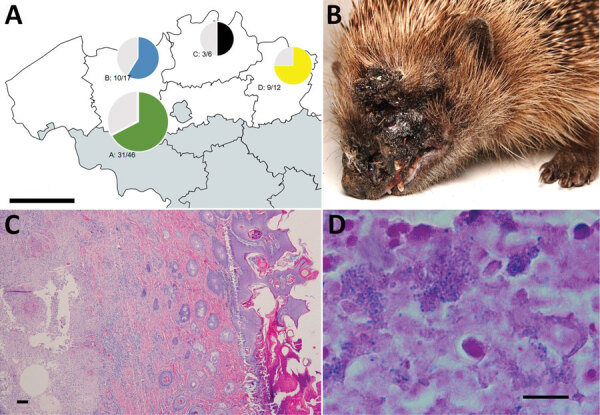
Toxigenic *Corynebacterium ulcerans* as cause of widespread disease in hedgehogs (*Erinaceus europaeus*), Flanders, Belgium. A) Locations of 81 hedgehogs with lesions on the head or limbs from 4 regions in Flanders, Belgium, who were tested for *C. ulcerans*. Green, blue, black, and yellow indicate proportion of positive animals; gray indicates proportion of negative animals. Regions: A, Geraardsbergen; B, Merelbeke; C, Herenthout; D, Oudsbergen. Scale bar = 50 km. B) Representative clinical state with necrotizing facial dermatitis in 1 male hedgehog from Merelbeke. C) Ulcerative dermatitis with suppurative exudation and inflammation extending into the subcutis and underlying skeletal muscles. Hematoxylin and eosin stained; scale bar = 100 μm. D) Microcolonies of gram-positive bacilli in suppurative exudate. Gram stain; scale bar = 10 μm.

During May and June 2020, we tested 81 hedgehogs (*Erinaceus europaeus*) that had ulcerative skin lesions and were provided by the public to 4 animal rescue centers across northern Belgium (Figure 1). Cases derived from 3 provinces in Flanders (East Flanders, Antwerp, and Limburg); total surface area of these provinces is 8,310 km^2^. All hedgehogs were individually housed, and we conducted sampling after euthanasia or natural death. We obtained 60 *Corynebacterium ulcerans* isolates from ulcers or abscesses on the head or limbs from 53 of 81 investigated hedgehogs; all were adult males. For 6 animals, we obtained >1 isolate from different lesions.

Although *C. ulcerans* was isolated most often, lesions yielded abundant, polybacterial growth (Table 1). We showed by systematic postmortem examinations that 9 animals had a good body condition generally but had multiple cutaneous ulcers on the head and limbs. Histopathologic analysis of skin of these animals showed subacute, extensive, ulcerative dermatitis and suppurative exudation and crusting. Inflammation sometimes extended to the subcutis and even to underlying skeletal muscles.

**Table 1:** Bacterial isolates obtained from 81 diseased hedgehogs, Flanders, Belgium*

Bacterial species obtained from lesions	No. positive animals
*Corynebacterium ulcerans*	53
*Staphylococcus aureus*	25
*Enterococcus faecalis*	23
*Streptococcus canis*	18
*Streptococcus dysgalactiae*	18
*Proteus vulgaris/P. hauseri*	17
*Staphylococcus rostri*	15
*Proteus mirabilis*	14
*Streptococcus pyogenes*†	13
*Staphylococcus xylosus*	10
*Staphylococcus microti*	8
*Staphylococcus sciuri*	8
*Vagococcus fluvialis*	8
*Enterococcus avium*	7
*Escherichia coli*	7
*Staphylococcus pettenkoferi*	7
*Morganella morganii*	6
*Staphylococcus fleurettii*	6
*Pasteurella multocida*	4
*Corynebacterium amycolatum*	3
*Bacteroides fragilis*	3
*Staphylococcus simulans*	3
*Trueperella pyogenes*	3
*Vagococcus lutrae*	2
*Arcanobacterium haemolyticum*	1
*Bacillus* sp.	1
*Bacteroides pyogenes*	1
*Corynebacterium confusum*	1
*Corynebacterium rouxii*	1
*Enterococcus hirae*	1
*Enterobacter hormaechei*	1
*Gemella haemolysans*	1
*Lactococcus garvieae*	1
*Streptococcus gallinaceus*	1
*Streptococcus thoraltensis*	1

*Bacteria were identified by using matrix-assisted laser desorption/ionization time-of-flight mass spectrometry. Biovar identification for the *C. diphtheriae* isolate was performed by using the Api Coryne System (bioMérieux, https://www.biomerieux.com). †Also detected by Berger et al. (*1*) and Franklinos et al. (*2*).

In some instances, we observed nodular inflammation consisting of central necrosis admixed with degenerated neutrophils and bordered by a small rim of macrophages (abscess formation) and fistulation. We observed intralesional microcolonies of gram-positive bacilli. We subjected organs that showed macroscopic abnormalities to histopathologic analysis. Four animals had interstitial pneumonia, 1 animal had ascending hepatitis, and 1 animal had fibrinosuppurative epicarditis and intralesional gram-positive bacteria.

Despite presence of parasites related to skin disease (fly maggots; myiasis, n = 5; *Sarcoptes scabiei*, n = 1; and *Caparinia* spp., (n = 1) and pathogens related to systemic disease (herpesvirus, n = 2 [*3*] and lungworms; *Crenosoma striatum*, n = 2), we found no consistent evidence for other causes of primary disease. Although evidence is insufficient to conclusively attribute the observed lesions to *C. ulcerans*, its widespread and high-level occurrence in diseased male hedgehogs is a serious concern, given frequent exposure of humans to hedgehogs and because *C. ulcerans* is the predominant cause of human diphtheria in many countries in Europe (*4*).

**Figure 2:**
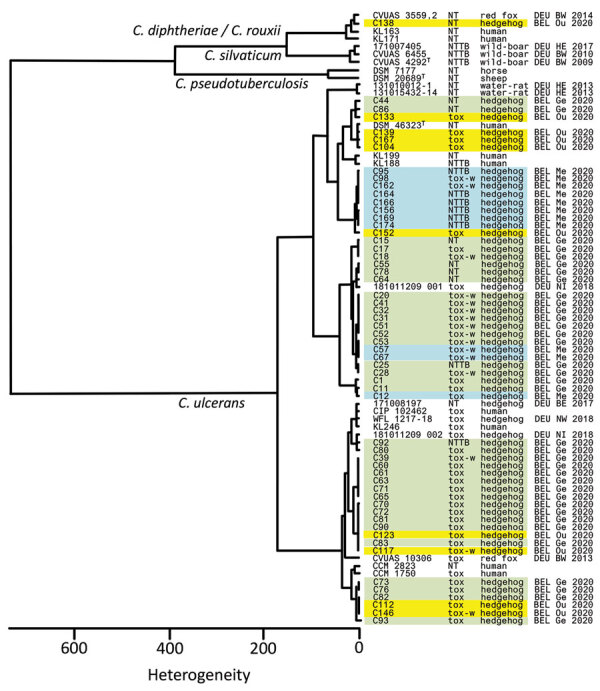
Dendrogram of Fourier-transformed infrared spectra of *Corynebacterium* spp. strains obtained from hedgehogs (*Erinaceus europaeus*), Flanders, Belgium, compared with spectra from several *C. ulcerans* isolates, including isolates from free-roaming red foxes (*Vulpes vulpes*) and wild boars *(Sus scrofa scrofa*) (*1*) and other well characterized and available isolates from animals and humans. Additional details for isolates were determined by using matrix-assisted laser desorption/ionization time-of-flight mass spectrometry (*8*). Country of origin: BEL, Belgium (region: Ge, Geraardsbergen [green]; Me, Merelbeke [blue]; Ou, Oudsbergen [yellow]); BE, Berlin; BY, Bavaria; DEU, Germany. NT, no tox gene; NTTB, nontoxic but tox-bearing (these are isolates that have the toxin gene, but do not produce toxins); T, type strain; Tox, toxigenic; tox-w, toxigenic (weak).

*C. ulcerans* isolates from hedgehogs belong to several clusters. We identified 56 isolates of *C. ulcerans* to the species level by using matrix-assisted laser desorption/ionization time-of-flight mass spectrometry (*5*) and sequencing of the *rpoB* gene (*6*). We also typed isolates by analysis of infrared spectra (*7*). Isolates grouped with *C. ulcerans* strains from humans and other animals (hedgehogs and red foxes [*Vulpes vulpes*] from Germany) (*1*) and clustered in 3 sublineages (Figure 2). The high diversity is similar to that reported by Berger et al. (*1*), and results argue against nocosomial infections and emergence and spread of a single *C. ulcerans* clone in the hedgehog population in Flanders. Instead, the high diversity of the isolates suggests *C. ulcerans* endemicity in the hedgehog population.

We found limited acquired antimicrobial resistance in *C. ulcerans* isolates from hedgehogs. We compiled MIC data for all *C. ulcerans* isolates (Table 2). Acquired resistance against enrofloxacin was detected in 4 isolates.

**Table 2:** Distribution of MIC values for 60 isolates of *Corynebacterium ulcerans* isolates from hedgehogs, Flanders, Belgium*

Drug	MIC, µg/mL											
	<0.016	0.016	0.032	0.064	0.12	0.25	0.5	1	2	4	8	16
Amoxicillin	1	10	6	8	25	10	0	0	0	0	0	0
Amoxicillin/clavulanate	2	2	2	12	18	22	2	0	0	0	0	0
Clindamycin	0	0	0	0	0	0	0	34	26	0	0	0
Doxycycline	0	0	0	0	5	27	13	13	2	0	0	0
Enrofloxacin	0	4	33	19	0	0	0	4	0	0	0	0
Erythromycin	0	1	50	9	0	0	0	0	0	0	0	0
Penicillin	1	1	4	6	17	20	11	0	0	0	0	0
Spiramycin	0	0	0	43	17	0	0	0	0	0	0	0
Tetracycline	0	0	0	0	0	11	29	19	1	0	0	0
Tilmicosin	0	0	0	0	0	0	1	29	29	1	0	0
Trimethoprim/sulfamethoxazole	0	0	9	43	8	0	0	0	0	0	0	0

*Values are no. isolates, which were obtained by using an epsilometer (ETEST; bioMérieux, https://www.biomerieux.com) on Mueller–Hinton agar plates containing 5% horse blood and 20 mg/L of β-NAD (bioMérieux), according to European Committee on Antimicrobial Susceptibility Testing (*9*) and Clinical and Laboratory Standards Institute (*10*) guidelines.

Most *C. ulcerans* isolates from hedgehogs produce toxins. We evaluated presence and expression of toxins by detection of the diphtheria toxin gene (*toxE*) by using a duplex PCR (*6*) and the Elek test (*11*). Results showed a positive result for this gene in 50/56 isolates by PCR and positive (26/56 isolates) or weak positive (16/56 isolates) results by Elek test. One animal was positive for the *toxE* gene in 1 location (*C. ulcerans* isolate from a head lesion) and negative for the gene in another location (isolate from a foot lesion). 

Although diphtheria vaccination coverage in humans is high in Belgium, since 2010, sporadic cases (14 cases during 2010–2017) of infection by toxigenic corynebacteria have occurred (*6*). Because presence of the *toxE* gene and toxin production are associated with pathogenicity in humans, these results suggest a zoonotic potential of most hedgehog-derived *C. ulcerans* isolates.

## Conclusions

Hedgehogs are mammals that are abundant in Europe and are frequently observed in nature reserves and urbanized areas. Because of their defensive behavior, sick animals are easily brought to animal rescue centers by the public, as testified by the large number of animals we examined in a short time frame during this study. The nature of their spiny defense promotes breaching and inoculating of the human epidermis with bacteria during handling. Other potential routes of transmission might include bite wounds or contact with the contaminated environment of the hedgehogs. Several potentially zoonotic or anthroponotic bacterial species, including *C. rouxii* and *Streptococcus pyogenes*, are associated with ulcerative lesions in diseased hedgehogs (Table 1).

Although Gower et al. (*12*) showed that the major risk factor for *C. ulcerans* infection in humans is exposure to domestic animals (e.g., dogs, cats), widespread occurrence of toxigenic *C. ulcerans* in most diseased hedgehogs across Flanders should prompt authorities to alert all stakeholders, including members of the public and staff at animal rescue centers, to take precautionary measures when handling hedgehogs. Although vaccination against *C. diphtheriae* protects against *C. ulcerans* disease, exposure to *C. ulcerans* from susceptible persons might result in severe disease (*12*). Recommendations should include wearing protective gloves and cleaning and disinfecting hands and fomites after contact with a hedgehog, as well as vaccination of persons who are frequently exposed to hedgehogs.

Treatment of infections with pyogenic coryneform bacteria in animals is challenging, and the 4 rescue centers involved in this study reported poor treatment success. Results of antimicrobial susceptibility testing suggest that this finding is not caused by acquired antimicrobial drug resistance but probably by insufficiently high antimicrobial drug concentrations reaching the *C. ulcerans* bacteria inside pus. Therefore, debrideing the lesions should be included in any treatment. Euthanasia should be considered for severe cases.

Emergence of *C. ulcerans* infection in hedgehogs is consistent with an increasing number of reports of *C. ulcerans* infections in wildlife across Europe and warrants attention across the continent (*1**,**13**,**14*). Reports dating back from the 1950s and the presence of several distantly related clusters of *C. ulcerans* in this study argue against a recent introduction of this pathogen in wildlife populations in Europe and favors the hypothesis that the observed and previously unreported high numbers of diseased hedgehogs result from pathogen emergence from a disease-endemic state. Although most wild animals affected with *C. ulcerans* have systemic infections (*1*), in our study, the manifestations of cutaneous disease dominated.

The finding that only male hedgehogs had this disease and that lesions are found mostly on body parts not covered with spines suggests the *C. ulcerans* infections might be opportunistic infections of wounds, arising from male-specific behavior during the mating season. Bite wounds are well known to be susceptible to infection with opportunistic pathogens that are part of the oral microbiota (*15*). Strain typing suggests that hedgehogs are a major reservoir of highly diverse *C. ulcerans* isolates. Active surveillance should elucidate the magnitude of this reservoir in healthy hedgehogs and the impact on the population level. Until the mechanisms underpinning the observed emergence of this potentially zoonotic wildlife disease from its disease-endemic state can be clarified, persons handling hedgehogs should take precautions to prevent possible transmission of *C. ulcerans*.
